# Effects of N-Glycan Composition on Structure and Dynamics of IgG1 Fc and Their Implications for Antibody Engineering

**DOI:** 10.1038/s41598-017-12830-5

**Published:** 2017-10-04

**Authors:** Hui Sun Lee, Wonpil Im

**Affiliations:** Departments of Biological Sciences and Bioengineering, Lehigh University, 111 Research Drive, Bethlehem, PA 18015 USA

## Abstract

Immunoglobulin G1 (IgG1), a subclass of human serum antibodies, is the most widely used scaffold for developing monoclonal antibodies to treat human diseases. The composition of asparagine(N)297-linked glycans can modulate the binding affinity of IgG1 Fc to Fc γ receptors, but it is unclear how the structural modifications of N-glycan termini, which are distal from the binding interface, contribute to the affinity. Through atomistic molecular dynamics simulations of a series of sequentially truncated high-mannose IgG1 Fc glycoforms, we found that the C′E loop and the Cγ2-Cγ3 orientation are highly dynamic, and changes in N-glycan composition alter their conformational ensembles. High-mannose glycoform preferentially samples conformations that are more competent to FcγRIIIa binding, compared to the truncated glycoforms, suggesting a role of IgG1 Fc N-glycan in optimizing the interface with the Fc receptor for efficient binding. The trajectory analyses also reveal that the N-glycan has large amplitude motions and the carbohydrate moiety interconverts between Fc-bound and unbound forms, enabling enzymatic modification of the glycan termini.

## Introduction

Antibody, also known as immunoglobulin (Ig), is produced by B cells and specifically binds to invading pathogens in the blood to stop them from functioning. Some antibodies play their role simply through the binding of their antigen-binding Fab domains to the target epitopes to block or induce signal transduction. On the other hand, many other antibodies recognize the antigen and then recruit circulating lymphoid and myeloid cells to kill the target through antibody-mediated effector functions (i.e., complement-dependent cytotoxicity, antibody-dependent cell-mediated cytotoxicity, and antibody-dependent cellular phagocytosis)^1–3^. Aside from these natural functions, its role as a bridge between pathogens and the immune system has made antibodies the fastest growing class of new drugs to treat a wide range of human diseases^3–5^.

**Figure 1:**
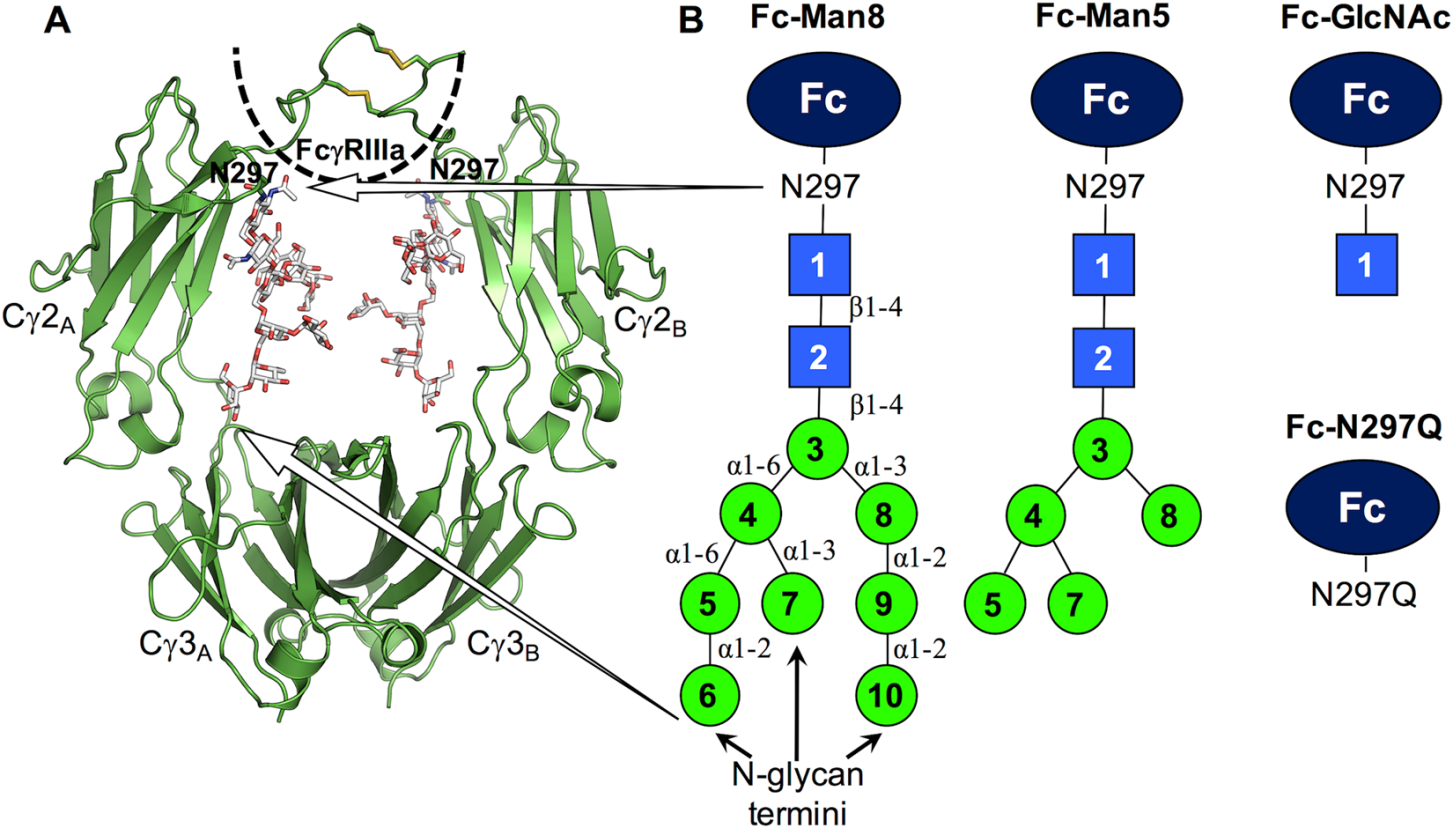
Structure of IgG1 Fc and its different glycoforms used in this study. (**A**) Initial structure of Fc-Man8. The IgG1 Fc dimer and N-glycans are shown in cartoon and stick representation, respectively. The black dotted line roughly illustrates FcγRIIIa. (**B**) Cartoons show IgG1 Fc glycoforms used in this study. Both Fc polypeptides have identical glycans or are aglycosylated except for Fc-Man8/N297 in which one Fc polypeptide chain is glycosylated by a high-mannose glycan, but the other aglycosylated. Key: *blue* square: *N*-acetylglucosamine, *green* circle: mannose.

Immunoglobulin G1 (IgG1) is a subclass of human serum antibodies and is the most widely used platform for developing therapeutic monoclonal antibodies^6^. The antibody-mediated effector functions require binding of the crystallizable fragment (Fc) of IgG1 to Fc γ receptors (FcγRs) that are expressed on the surface of recruited cells. IgG1 Fc is a symmetric homodimer comprised of the C-terminal half of Ig heavy-chain polypeptides, and each consists of an N-terminal Cγ2 domain and a C-terminal Cγ3 domain (Fig. 1A). The homodimer is formed by disulfide bonds between the N-terminal hinge regions in the Cγ2 domains and non-bonded interactions between the Cγ3 domains.

Glycosylation, the most common post-translational modification, and its effects on protein structure and function have been a topic of interest for many years^7–10^. Many experiments have provided evidences that glycosylation can alter thermodynamic, kinetic, and structural features of proteins, resulting in modulation of a broad array of biological processes such as protein folding, stability, oligomerization, quality control, trafficking, enzyme activity, and host cell-surface interactions^11–14^. We previously performed a Protein Data Bank (PDB) survey and a molecular dynamics (MD) simulation study to investigate the common effects of asparagine (N)-linked glycans on protein structure and dynamics. Our study indicates that the glycosylation does not induce significant changes in protein structure, but lowers structural fluctuation^8^. Given a specific protein, however, effects by glycosylation become more sophisticated. In the case of IgG1, the binding of its Fc domain to FcγRs requires the post-translational modification of the Fc region with an N-glycan at Asn 297^15,16^. In addition, composition changes in the IgG1 Fc glycans are also known to affect the binding affinity^17–20^.

The crystal structures of IgG1 Fc in complex with an Fc receptor, FcγRIIIa, have revealed that a significantly better binding affinity of afucosylated IgG1 Fc to FcγRIIIa results from additional carbohydrate-carbohydrate interactions between the Fc and receptor glycans^21^. Particularly, it is noteworthy that changes in the N-glycan non-reducing termini, which are distal from the binding interface between IgG1 Fc and Fc*γ*Rs (Fig. 1), can modulate the binding affinity. However, a molecular-level understanding on how the structural modifications of N-glycan termini contribute to changes in Fc:Fc*γ*R binding affinity remains enigmatic. In addition to the biological significance of elucidating the role of N-glycans in Fc binding to Fc*γ*Rs, proper N-glycan remodeling is also required for the development of next generation IgG1-based antibodies through Fc N-glycan optimization^22–24^.

To explore both challenges (antibody biology and engineering), we investigated the impacts of different N-glycan compositions on the structure and dynamics of IgG1 Fc using atomistic MD simulations of a series of sequentially truncated high-mannose IgG1 Fc glycoforms (Fig. 1B): (1) Fc with high-mannose N-glycans (Man_8_GlcNAc_2_, Fc-Man8), (2) Fc with five mannoses (Man_5_GlcNAc_2_, Fc-Man5), (3) Fc with a single core *N*-acetylglucosamine (Fc-GlcNAc), (4) aglycosylated Fc (Fc-N297Q), and (5) Fc with an asymmetric glycoform (Fc-Man8/N297Q). These simulations collectively reveal that IgG1 Fc is highly dynamic and structural changes in glycan termini modulate a conformational ensemble of IgG1 Fc. High-mannose glycoform preferentially samples C′E loop conformation and Cγ2-Cγ3 orientation that are most relevant to FcγRIIIa binding, compared to the truncated glycoforms. We also report large amplitude motions of N-glycans, causing most carbohydrate moieties to be detached from the Fc polypeptide surface and making them accessible for enzymatic modifications of the glycan termini.

## Results

### Glycans interconvert between Fc-bound and unbound forms

**Figure 2:**
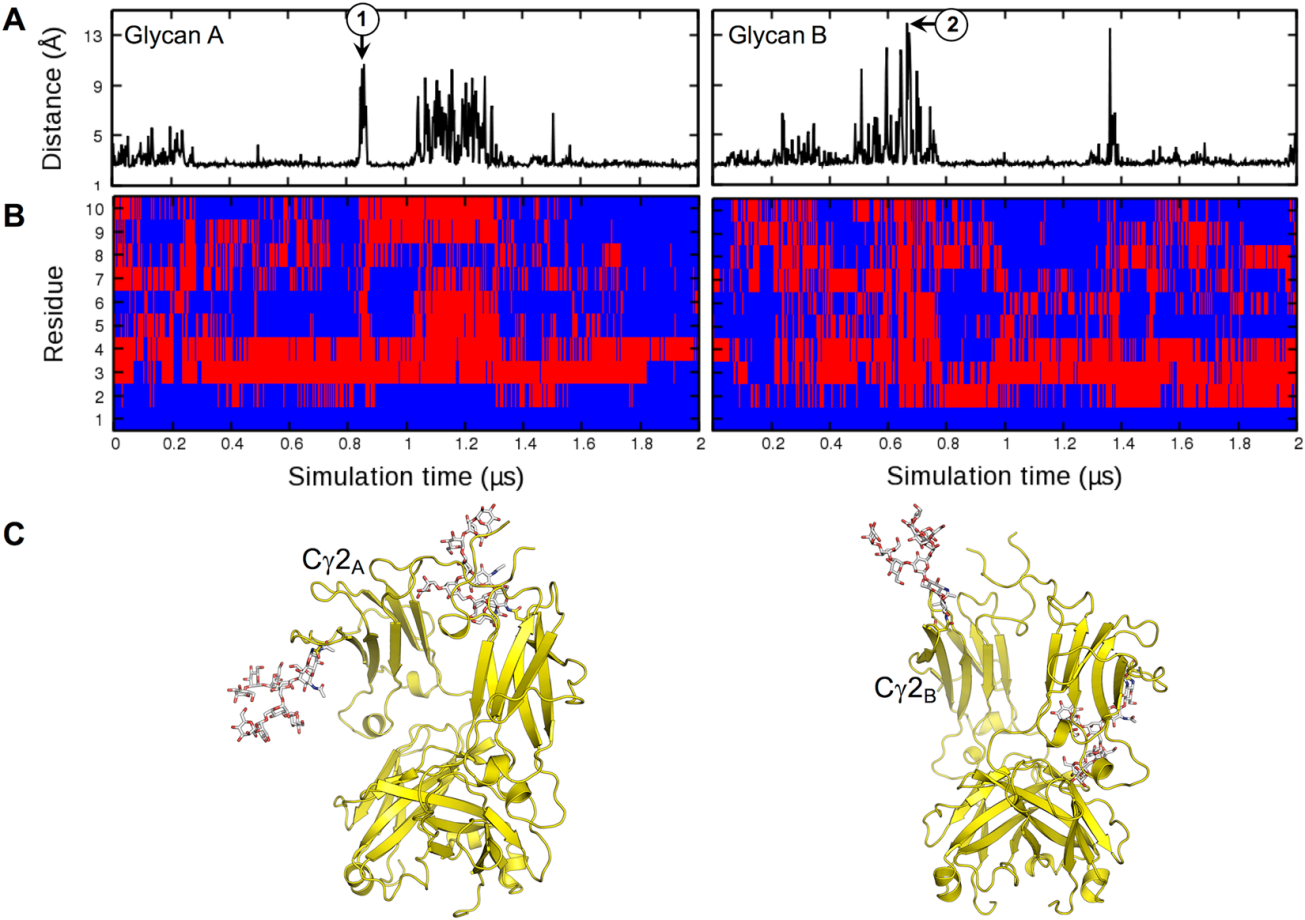
N-glycan motions in Fc-Man8. (**A**) Minimum distances between any heavy atoms of glycan termini (residues 6, 7, and 10 in Fig. 1B Fc-Man8) and any protein heavy atoms during the simulation. (**B**) The existence of heavy atom contacts (distance cutoff = 4.5 Å) between each glycan residue and IgG1 Fc during the simulation. If there are contacts, it is colored blue. Otherwise, it is colored red. (3) Representative structures (snapshots 1 and 2) chosen at the simulation times (marked in (**A**)) with the maximum distances for each glycan.

**Figure 3:**
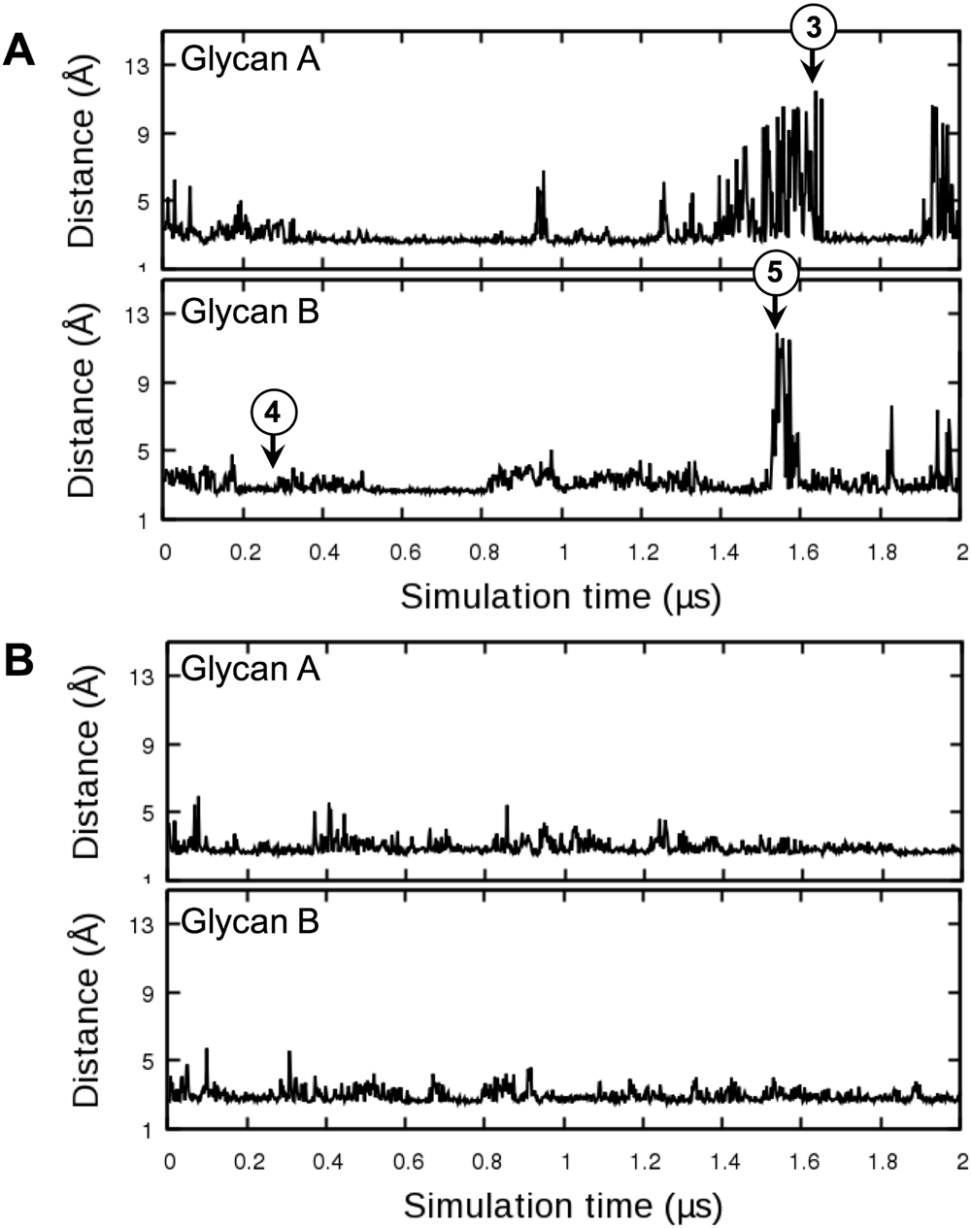
N-glycan motions in Fc-Man5 and Fc-Man8_R. (**A**) Minimum distances between any heavy atoms of glycan termini (residues 5, 7, and 8 in Fig. 1B Fc-Man5) and any protein heavy atoms. (**B**) Minimum distances in Fc-Man8_R that is an independent replica simulation of Fc-Man8.

To characterize the dynamics of Fc N-glycans during the simulations, we measured minimum distances between any heavy atoms of the terminal residues of each glycan (e.g., mannoses 6, 7, and 10 in Fig. 1B Fc-Man8) and any protein heavy atoms as a function of simulation time (Fig. 2A). Many peaks with distances > 4.5 Å are shown in the time-series, indicating that the glycan termini have no contact with the Fc surface and they are exposed to the bulk water (Fig. 2B). The sharp minimum distance peaks (i.e., minimum distance > 9 Å) correspond to the cases where most glycan residues are detached from the Fc surface. The representative structures are illustrated in Fig. 2C. Such large amplitude motions are also observed for Fc-Man5 (Fig. 3A). We performed an additional 2-μs simulation as an independent replica for Fc-Man8 (Fc-Man8_R) to further examine the large-amplitude glycan motions. In this simulation, however, the minimum distance time-series (Fig. 3B) do not show the complete detachment of either glycan. Collectively, our simulations indicate that Fc glycans have highly dynamic motions enabling interconversion between Fc-bound and unbound conformational states, but such motions are stochastic. Supplementary Video S1–S6 show 2-µs simulation movies for five systems (Fc-Man8, Fc-Man5, Fc-GlcNAc, Fc-N297Q, and Fc-Man8/N297Q) and the independent replica of Fc-Man8 (Fc-Man8_R).

### Highly fluctuating C′E loop is an innate property of IgG1 Fc

**Figure 4:**
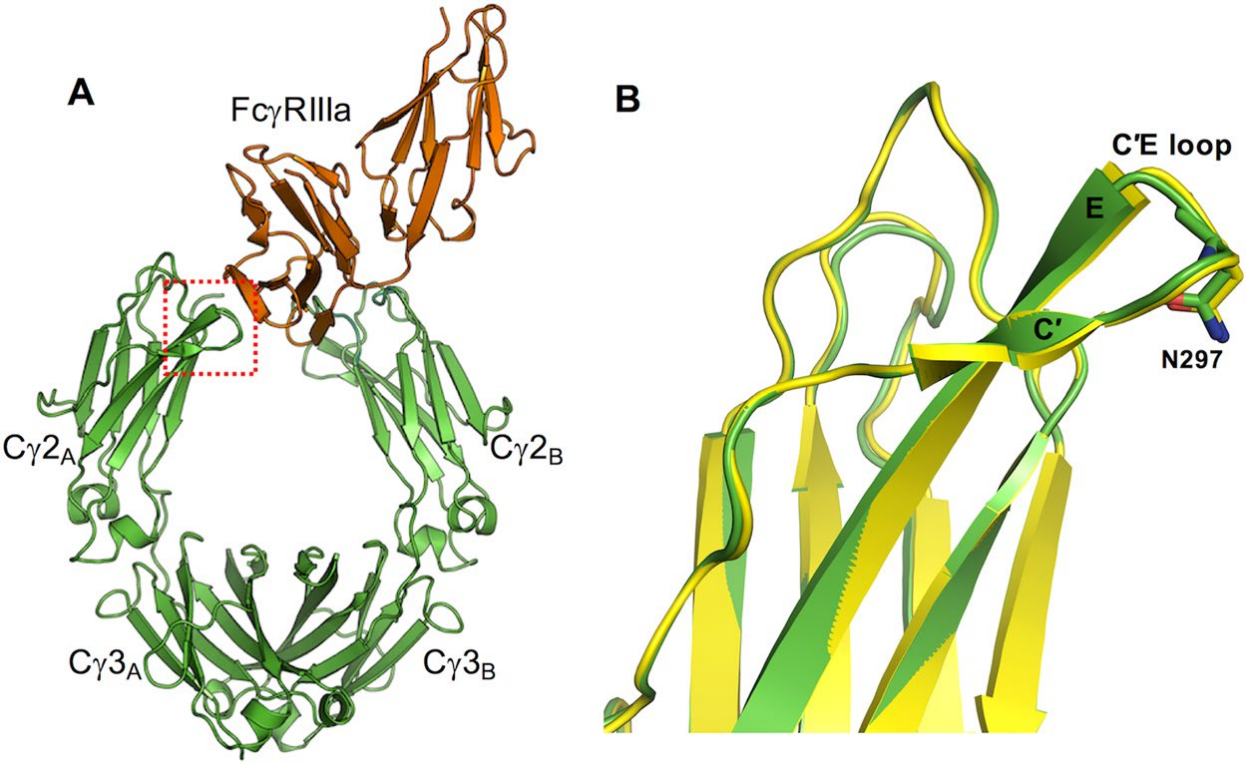
Structure of IgG1 Fc in complex with FcγRIIIa. (**A**) Cartoon representation of IgG1 Fc/FcγRIIIa complex (PDB: 3sgk). Red dotted box is shown to indicate the C′E loop region. (**B**) Structure comparison of C′E loop between the MD initial structure and IgG1 Fc/FcγRIIIa crystal structure (RMSD = 0.42 Å).

To investigate the effects of N-glycan composition on the local structure and dynamics of IgG1 Fc, we focus our analyses on a structural element in the Fc region, the so-called C′E loop (Gln295 –Tyr – Asn297 – Ser – Thr299; Fig. 4). The C′E loop not only contains the N-glycosylation site, but also is a primary interface involved in intermolecular interactions with FcγRIIIa (Figure S1A). Other studies have reported that the binding affinity of IgG1 Fc to FcγRIIIa is attributed to specific conformations and/or structural fluctuations of the C′E loop^25,26^. The initial simulation structures have a C′E loop conformation very similar to that in the crystal structure of IgG1 Fc:FcγRIIIa complex (PDB: 3sgk) (Fig. 4B and Figure S1B).

**Figure 5:**
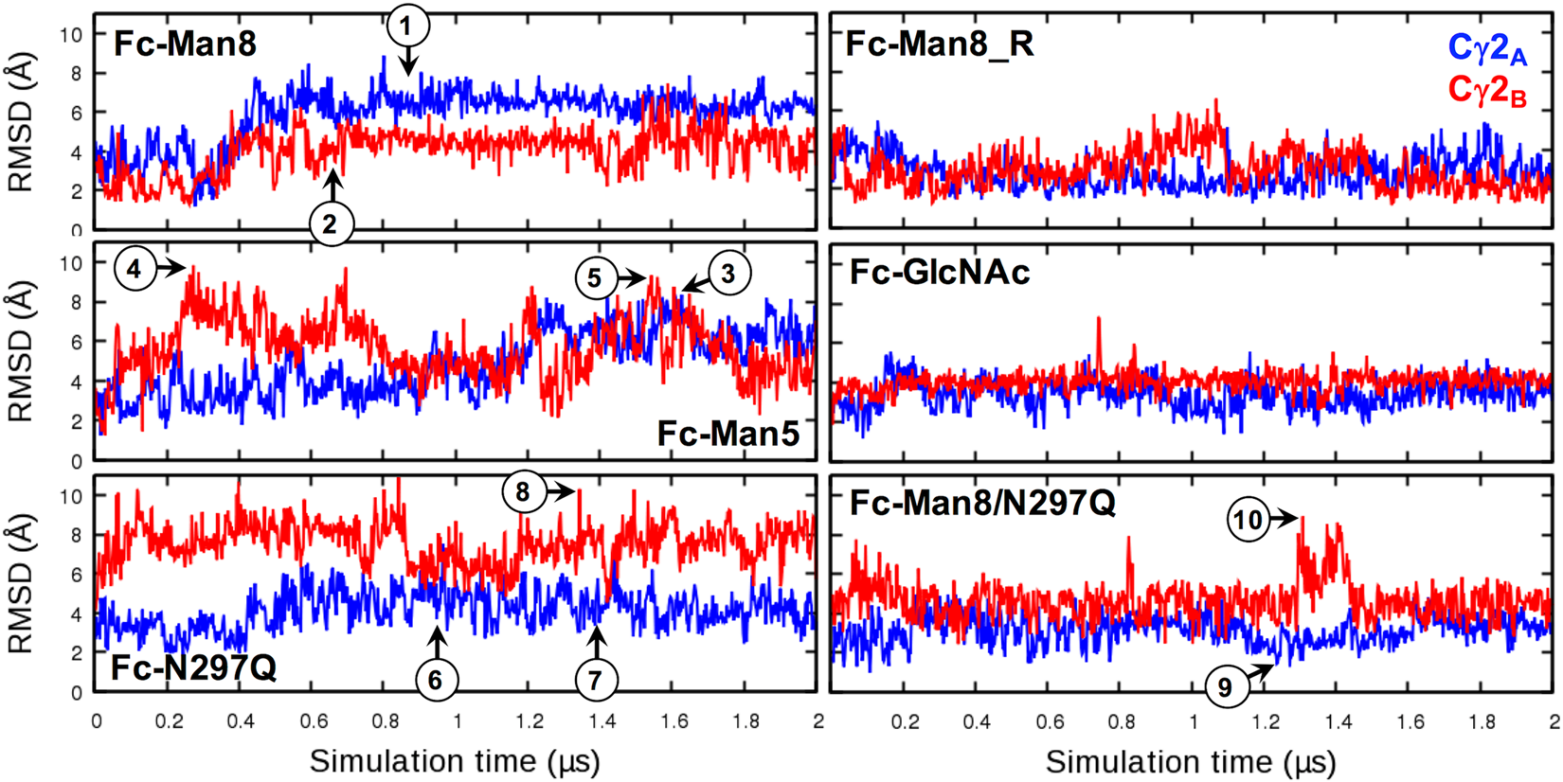
Root-mean-square deviation (RMSD) time-series of C′E loop. The RMSD was calculated using all heavy atoms of C′E loop with respect to the initial structure after superposing each snapshot onto the initial structure using Cα atoms of residues 259–265 and 301–306. The circled numbers on the plots are simulation times where representative structures are taken for structural comparison. Snapshots 1 to 5 are the same in Fig. 2A and Fig. 3A. The RMSD time-series for polypeptide A and B are colored blue and red, respectively.

**Figure 6:**
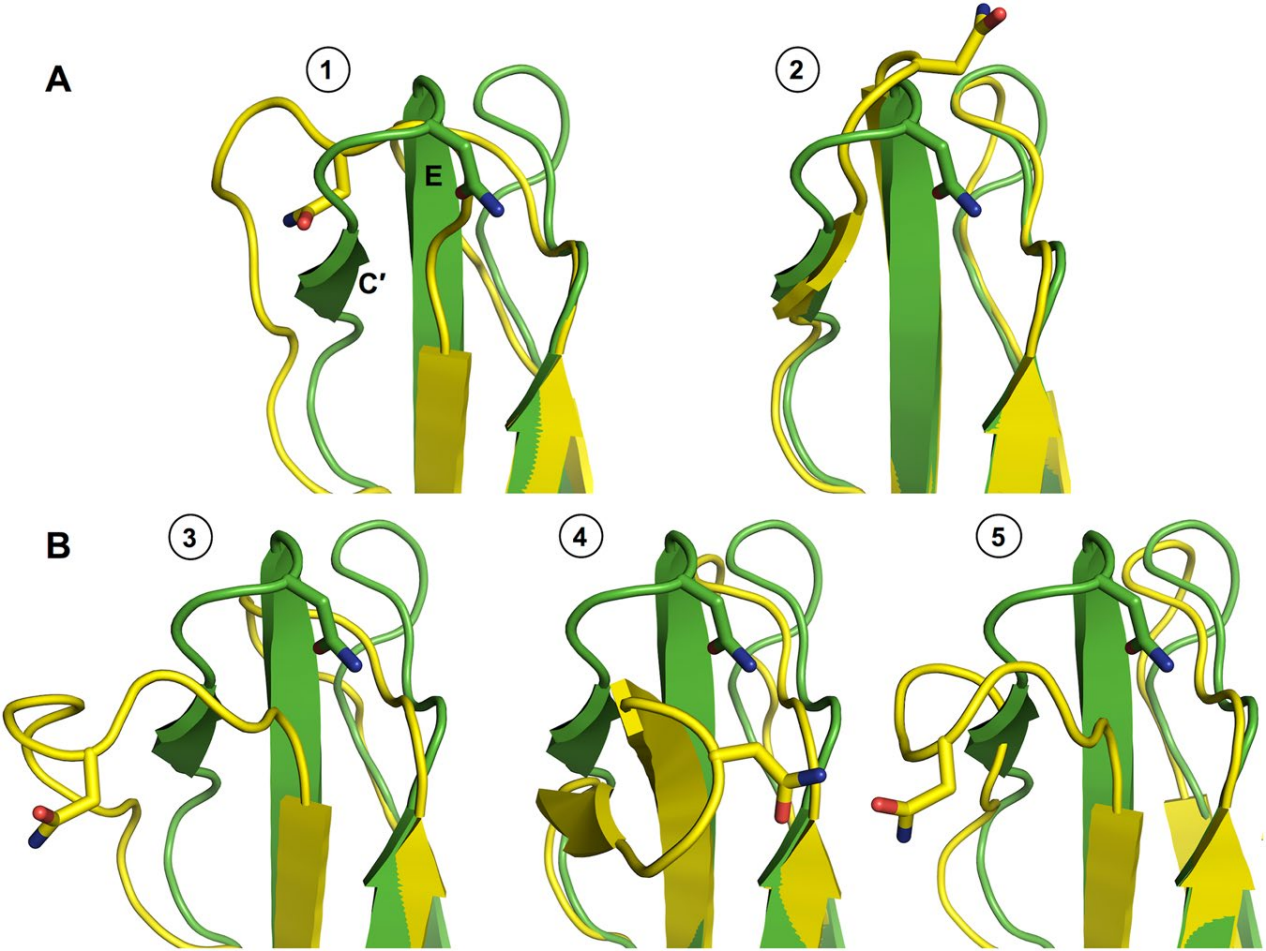
Comparison of representative MD structures (yellow) with the initial structure (green) for C′E loop region. (**A**) Snapshots 1 and 2 for Fc-Man8 polypeptide chain A and B, respectively. (**B**) Snapshots 3 for Fc-Man5 chain A and snapshots 4 and 5 for Fc-Man5 chain B.

Figure 5 shows the time-series of root-mean-square deviations (RMSDs) of the C′E loop with respect to the initial structure. To better understand a relationship between the RMSD profiles and the C′E loop conformations, we compared representative MD snapshots with the initial structures. We first examined the C′E loop conformations where N-glycan is fully detached from the Fc surface (i.e., snapshots 1 and 2 for Fc-Man8 and 3 and 5 for Fc-Man5; also see Fig. 2A and Fig. 3A for the minimum distance plots). The structural comparisons in Fig. 6 indicate that large conformational changes of C′E loop (with the RMSDs of 6.12 Å, 7.61 Å, and 8.32 Å for snapshots 1, 3, and 5, respectively) together with unstructured short C′ β-strand (Figure S2) are commonly accompanied with the glycan detachment. For the future reference, these C′E loop conformations are classified into an “unstructured state”.

In contrast to snapshots 1, 3, and 5, snapshot 2 has a relatively low RMSD (3.49 Å) and structured C′ strand, but the C′E loop has a less bent conformation, facing outside the cavity between the Fc polypeptide monomers. So, this conformation is classified into an “outward-facing state”. This structure also indicates that disordered C′ strand is not a necessary prerequisite for N-glycan to be detached from the Fc surface or a consequence of the glycan detachment. We then examined the snapshot 4 of Fc-Man5 in which no glycan detachment is observed in Fc polypeptide chain A (Fig. 3A), but the C′E loop RMSD is notably high (9.84 Å). In this case, the C′ strand is well ordered, but the C′E loop and its linked strands bends towards the Fc cavity. This conformation is classified into an “inward-facing state”.

**Figure 7:**
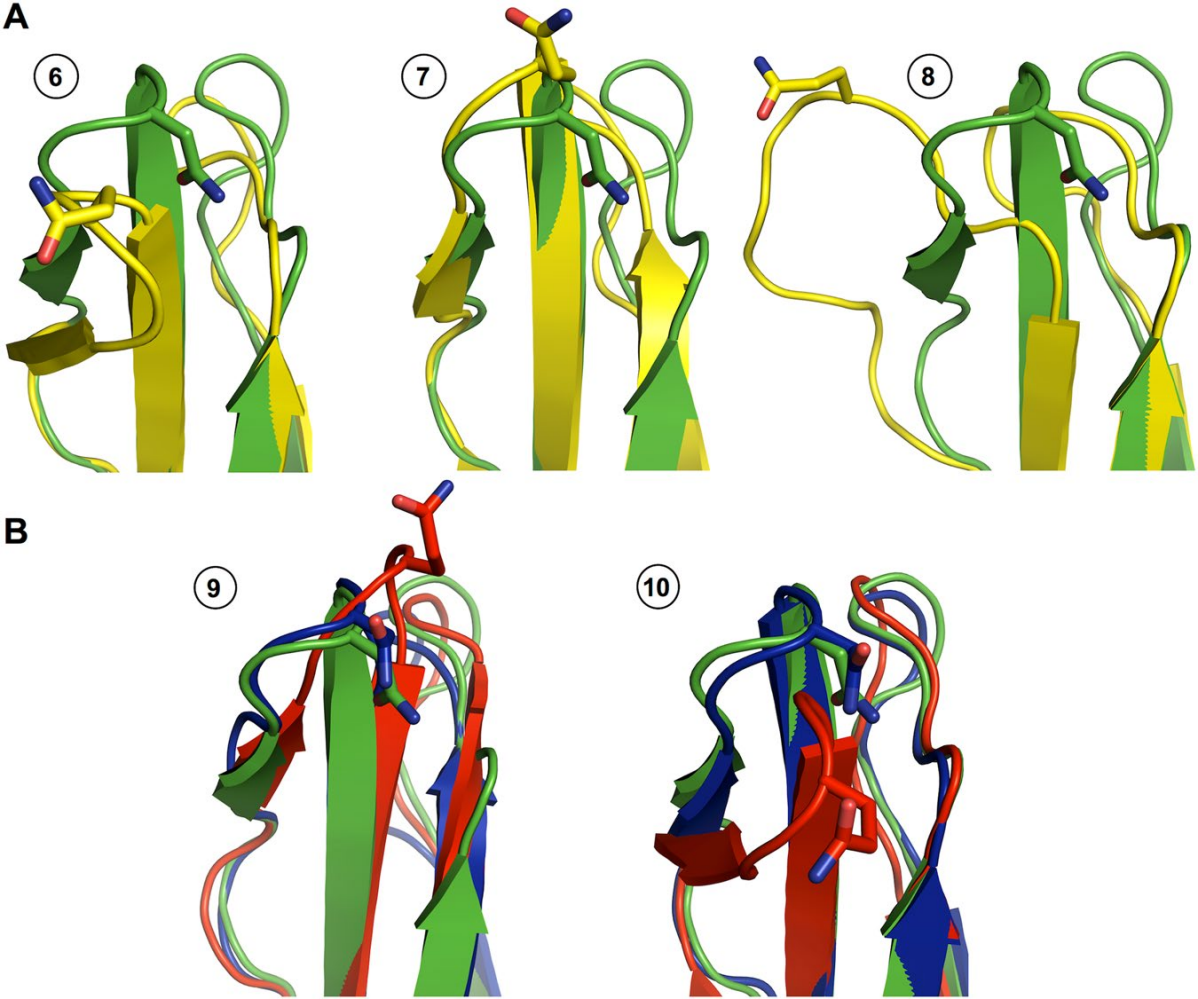
Comparison of representative MD structures with the initial structure for C′E loop region. (**A**) Snapshots 6 and 7 for Fc-N297Q polypeptide chain A and snapshot 8 for Fc-N297Q chain B. The initial structure and MD snapshots are colored green and yellow, respectively. (**B**) Snapshots 9 and 10 for Fc-Man8/N297Q. The initial structures are colored green. The structures for chain A and chain B are colored blue and red, respectively.

Based on these classifications, we examined if the C′E loop conformations sampled by aglycosylated form are different from those by glycosylated forms. To address this, three representative snapshots 6, 7, and 8 (marked in Fig. 5) are shown in Fig. 7A. The structural comparisons indicate that aglycosylated Fc sampled all different conformational states (i.e., snapshot 6: inward-facing state with an RMSD of 7.52 Å, snapshot 7: outward-facing state with an RMSD of 3.50 Å, and snapshot 8: unstructured state with an RMSD of 10.31 Å) in Fc-Man8 and Fc-Man5. The C′E loop conformational fluctuations between the outward- and inward-facing states are also clearly observed in the aglycosylated polypeptide in Fc-Man8/N297Q (Fig. 7B; snapshots 9 and 10 marked in Fig. 5). Collectively, our simulations indicate that the C′E loop conformation varies and the structural fluctuation results from innate dynamics of the Fc polypeptides, regardless of the existence of N-glycans.

### N-glycan composition alters an ensemble of C′E loop conformations

To characterize a difference in C′E loop conformations in between different Fc glycoforms, we analyzed the trajectories where C′E loop conformations are not related to the glycan detachment from the protein surface and unstructured C′ strand (i.e., the loop has relatively low RMSDs and remain stable over the entire simulation time). These trajectories are Fc polypeptide chains A and B in Fc-Man8_R, Fc-GlcNAc, and Fc-Man8/N297Q and chain A in Fc-N297Q. We calculated their average RMSDs during the last 1.5 μs and the values are 2.68 ± 0.76 Å (chain A) and 3.04±1.01 Å (chain B) in Fc-Man8_R, 3.40±0.67 Å (chain A) and 4.11±0.48 Å (chain B) in Fc-GlcNAc, 4.42±0.83 Å (chain A) in Fc-N297Q, and 3.13±0.68 Å (chain A) and 4.66±0.97 Å (chain B) in Fc-Man8/N297Q. The data show that, although the differences are subtle, the C′E loop in high-mannose IgG1 Fc glycoform least deviates from the initial structure and on average is most similar to the crystal structure of IgG1 Fc:FcγRIIIa complex. The structural deviation of the C′E loop becomes larger as the N-glycans are more truncated. It is noted that the asymmetric system, Fc-Man8/N297Q has clearly separated RMSD profiles between high-mannose glycosylated and aglycosylated polypeptide (Fig. 5). The representative snapshots taken at simulation time 9 and 10 are illustrated in Fig. 7B to show the difference in C′E loop conformations between the Fc polypeptides.

**Figure 8:**
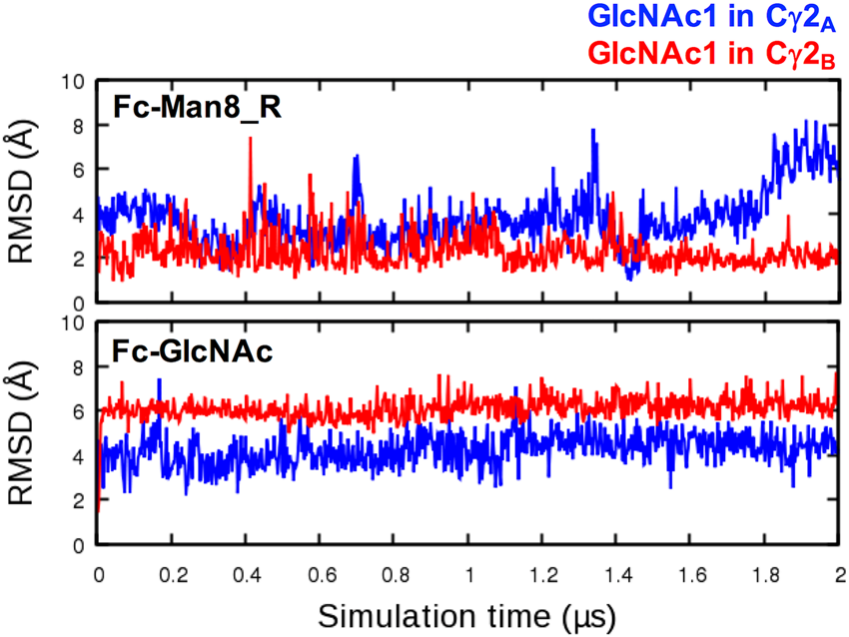
RMSD time-series of the first glycan core residue GlcNAc1 in Fc-Man8_R and Fc-GlcNAc. The RMSD was calculated using all heavy atoms of GlcNAc1 with respect to the initial structure after superposing each snapshot onto the initial structure using Cα atoms of residues 259−265 and 301−306. The RMSD time-series for GlcNAc1 in polypeptide A and B are colored blue and red, respectively.

The crystal structure of IgG1 Fc in complex with FcγRIIIa reveals that the first GlcNAc (GlcNAc1) of Fc forms direct hydrogen bonds with the first two GlcNAc units of the receptor, indicating significant contributions to the protein-protein binding affinity^21^ (Figure S1A). Our MD initial structures also have a conserved GlcNAc1 conformation as in the IgG1 Fc:FcγRIIIa crystal structure (Figure S1B). We measured the RMSD time-series of GlcNAc1 and calculated the average RMSD during the last 1.5 μs (Fig. 8). The values are 3.85±1.32 Å and 2.26±0.68 Å for Fc-Man8_R and 4.37±0.66 Å and 6.15±0.46 Å for Fc-GlcNAc, showing that the conformational changes of the C′E loop also lead to structural and orientational deviation of GlcNAc1. Taken together, the results suggest that a high-mannose glycan stabilizes the C′E loop and the GlcNAc1 into a conformation that is more competent to Fc receptor binding, compared to the truncated ones.

### N-glycan composition alters a conformational ensemble of Cγ2-Cγ3 orientation

**Figure 9:**
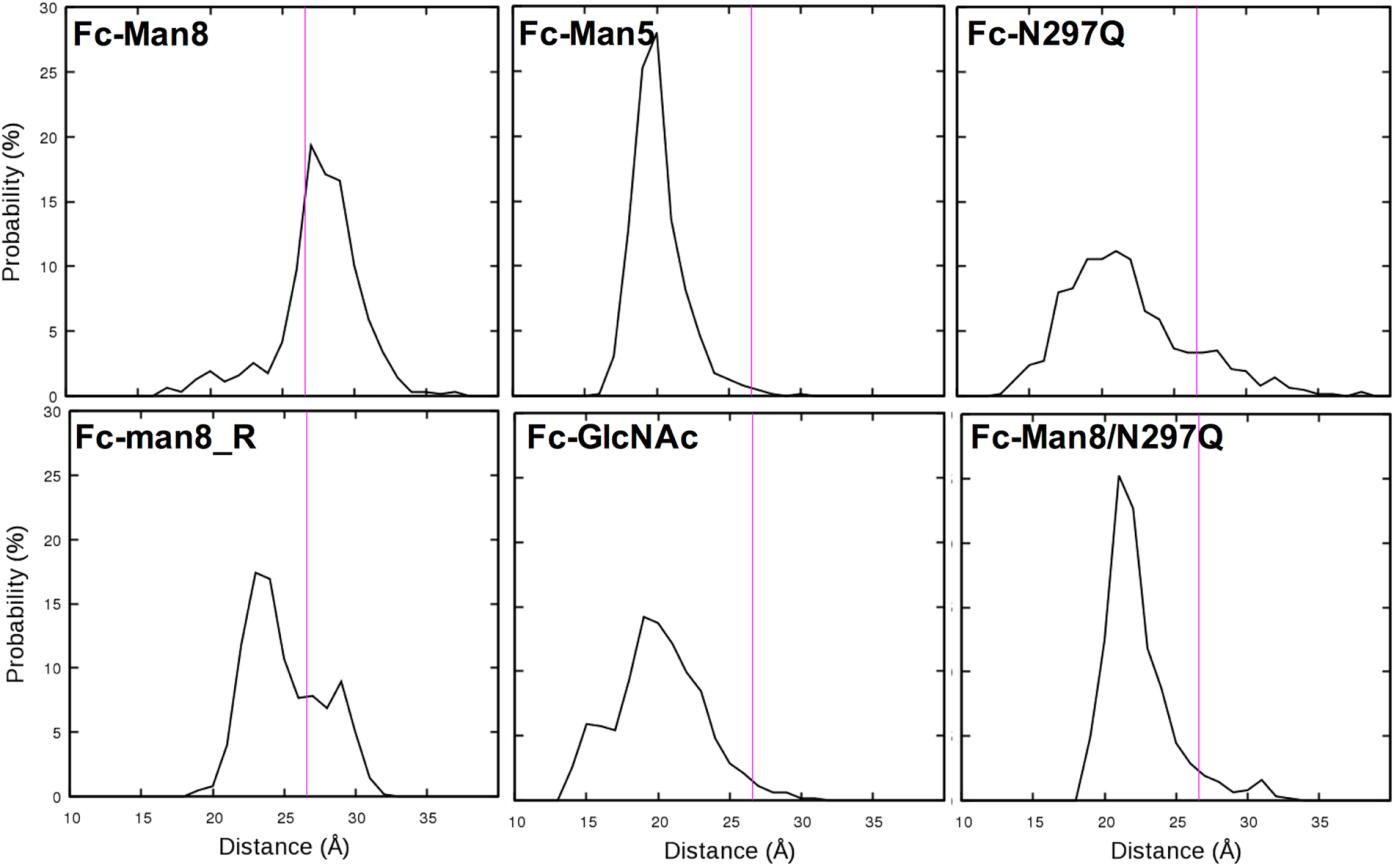
Distributions of distances between the geometric centers of Fc surfaces for FcγRIIIa binding. The magenta line in each plot corresponds to the distance in the crystal structure of IgG1 Fc: FcγRIIIa complex.

We examined if different N-glycan compositions affect the global motions of IgG1 Fc. We assigned FcγRIIIa-interacting Fc residues based on the IgG1 Fc/FcγRIIIa crystal structure and then measured the distances (*D*_AB_) between the geometric center of the chain A residues and that of the chain B residues. Their probability distributions calculated using the last 1.5-μs trajectories show that high mannose-containing systems (Fc-Man8 and Fc-Man8_R) sample significantly more Fc conformations whose *D*_AB_ are comparable to that in the crystal structure, whereas the *D*_AB_ in other systems are all biased to the smaller distance (Fig. 9).

**Figure 10:**
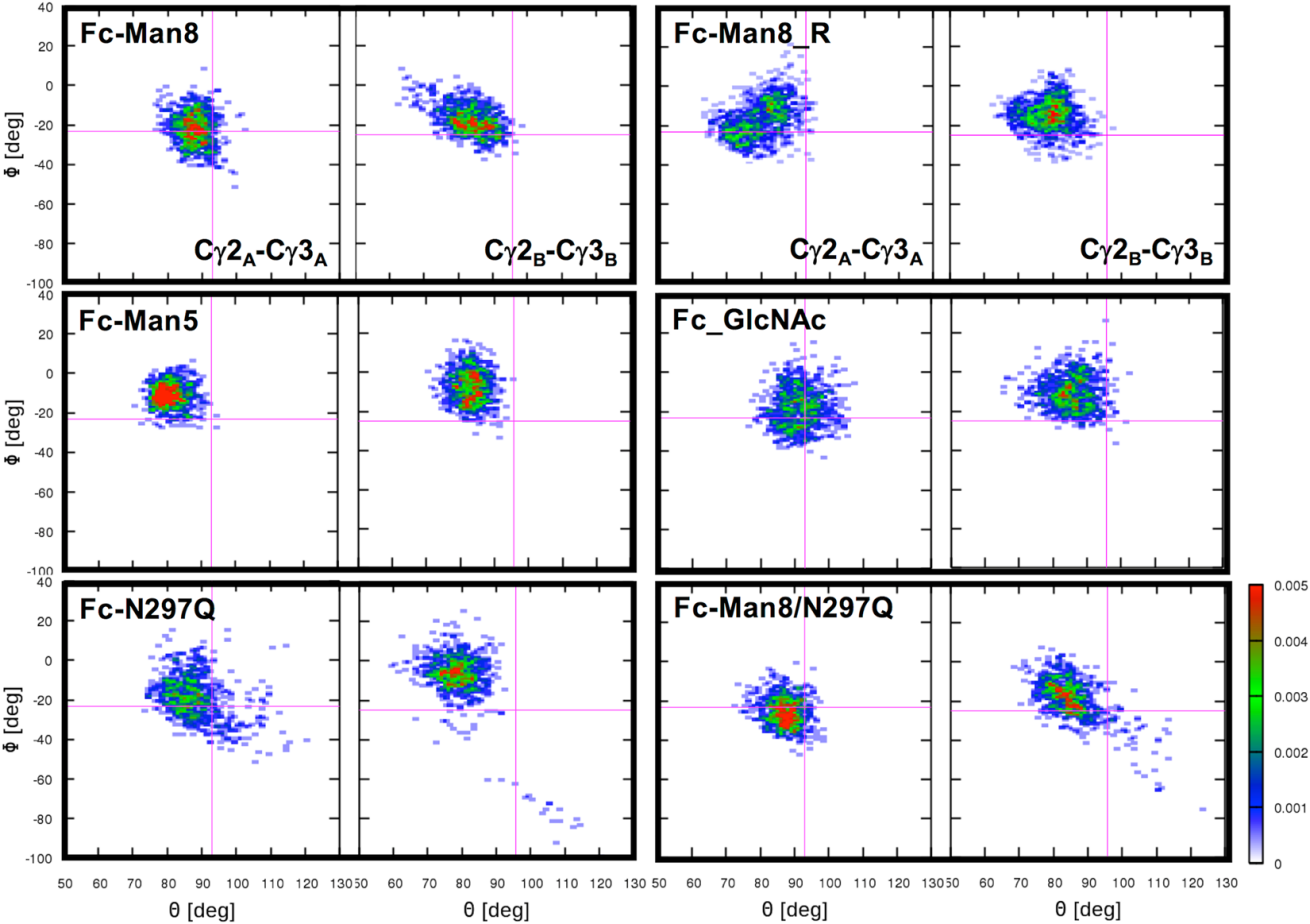
Distributions of Cγ2-Cγ3 angles (*θ*) and dihedral angles (*ϕ*). The definitions of *θ* and *ϕ* are shown in Figure S3. The magenta lines in each plot correspond to the angle and dihedral angle in the initial structure.

We also measured angles (*θ*) and dihedral angles (*ϕ*) between Cγ2 and Cγ3 in each Fc chain and between Cγ3 _A_ and Cγ3_B_ to investigate global conformational changes in IgG1 Fc; Figure S3 shows the points used to define *θ* and *ϕ*, which are based on Frank *et al*.’s study^27^. As shown in Fig. 10, the intra-chain Cγ2-Cγ3 motions are highly dynamic (e.g., *θ* = 68.0° to 104.0° and *ϕ* = −51.4° to 17.7° for Fc-Man8 chain A), while the inter-chain Cγ3-Cγ3 motions (Figure S4) were much more restricted (e.g., *θ* = 79.3° to 91.7° and *ϕ* = 105.7° to 127.4° for Fc-Man8), indicating stable inter-Cγ3 domain interactions to form a homodimer. It appears that the Cγ2-Cγ3 domain orientations do not show a significant correlation with the detachment of N-glycans from the Fc surface (Figure S5). A distinguishable feature from the distribution plots is that the ranges of Cγ2-Cγ3 *θ* and *ϕ* become markedly broader when Fc polypeptide chain is aglycosylated (i.e., chains A and B in Fc-N297Q and chain B in Fc-Man8/N297Q), suggesting that even the presence of a single monosaccharide can confer significant impact on Fc dynamics.

### Concluding Discussion

The data presented herein clearly show that the glycosylated local region (i.e., C′E loop) and Cγ2-Cγ3 domain orientation of IgG1 Fc are highly dynamic and different N-glycan compositions alter their structural ensembles. High-mannose glycoform preferentially samples the C′E loop conformations and the Cγ2-Cγ3 orientations that are more competent to FcγRIIIa binding, compared to truncated glycoforms. This strongly suggests that in addition to its direct interactions with the Fc receptors, a role of IgG1 Fc N-glycan is to optimize the interface with the Fc receptor for efficient binding. Our simulations also show that Fc N-glycan has dramatic motions in which the carbohydrate moiety is detached from Fc polypeptide surface, enabling enzymatic modification of the glycan termini.

Okbazghi *et al*. recently produced four different IgG1 Fc glycoforms (Fc-Man8, Fc-Man5, Fc-GlcNAc, and Fc-N297Q) as a model system and evaluated their binding affinity to FcγRIIIa^17^. Fc-Man8 and Fc-Man5 have a comparable biding affinity to the receptor (*K*_*D*_ ~ 26 nM for Fc-Man8 and ~32 nM for Fc-Man5), while Fc-GlcNAc has weak affinity (*K*_*D*_ ~ 995 nM), and Fc-N297Q does not show measurable affinity. Intriguingly, their experimental measurements correlate with the average C′E loop RMSDs from our simulations (i.e., the lower the average RMSD is, the higher the binding affinity is), suggesting that preorganized C′E loop conformation is a determinant of high binding affinity of IgG1 Fc to FcγRIIIa. As an additional experimental support, Subedi and Barb recently reported that the primary role of IgG1 Fc N-glycan is to restrict the C′E loop conformation for optimal binding affinity based on their solution nuclear magnetic resonance (NMR) spectroscopy data^26^. It is expected that the weakest affinity of aglycosylated form is also attributed to the absence of GlcNAc1 that is a key moiety for FcγRIIIa interactions.

The NMR study by Subedi and Barb also reported that the Cγ2-Cγ3 domain orientation of glycosylated Fc is indistinguishable from aglycosylated Fc, indicating that N-glycans little affect the global conformation of IgG1 Fc and, as a result, do not predominantly contribute to binding affinity to FcγRIIIa^26^. On the other hand, Borrok *et al*. found that the aglycosylated Fc has a larger radius of gyration (*R*_g_) than glycosylated Fc by small-angle X-ray scattering (SAXS), indicating that the aglycosylated form has more open Fc conformations^28^. Our simulations show that high-mannose and aglycosylated Fc have a comparable *R*_g_ (25.86±0.37 Å for Fc-Man8 and 25.60±0.58 Å for Fc-N297Q), but the distributions of Cγ2-Cγ3 angle and dihedral angle are markedly broad in the aglycosylated form, indicating high fluctuation of Cγ2-Cγ3 orientation (Fig. 9 and Fig. 10). Interestingly, the conformations of high-mannose glycoforms are more populated in a state where Fc binding interfaces with FcγRIIIa are more preorganized, whereas the distribution in the aglycosylated form is biased to a more closed binding interface state (Fig. 9). This suggests a possible role of N-glycan in maintaining IgG1 Fc in the conformation for optimal binding to the Fc receptors, even though it is hard to estimate how dominantly this effect contributes to the binding affinity.

Our simulations show great amplitude motions of Fc N-glycan in which most of the glycan residues are detached from Fc surface, exposing the glycan termini to the bulk solvent for enzymatic modification. This is consistent with studies on Fc glycan accessibility and NMR dynamics that Fc glycans have two distinct states: one with the glycan termini sequestered by interactions with the polypeptide surface and the other with glycan termini free from the glycan-polypeptide interactions and exposed to the bulk solution^29,30^. Frank *et al*. performed a 200-ns MD simulation study for a complex-type biantennary glycan linked to IgG1 Fc, reporting exchanging motions of the glycans between bound and unbound conformations. However, their simulations did not provide evidence of glycan’s excursion from the Fc cavity for enzyme modification of glycan termini^27^. Our 2-μs simulations demonstrate the large-amplitude events at the molecule level.

Our MD simulations offer new insights into the motions of Fc glycans and the structure and dynamics of IgG1 Fc to better understand biological roles of Fc glycan. The data also highlight that our analyses (e.g., changes in C′E loop conformation and Cγ2-Cγ3 orientation) can be used as effective descriptors to predict binding affinity of IgG1 Fc to its receptors and eventually could guide engineering of IgG1-based antibodies through Fc N-glycan optimization for better efficiency.

## Methods

An initial structure of IgG1 Fc was generated using PDB:2wah because this IgG1 Fc has high mannose-type N-glycans and an open conformation. The original N-glycans consists nine Mannoses and two *N*-acetylglucosamines, but many residues are missing in the crystal structure (glycan residue 10 in chain A and residues 4–10 in chain B; see Fig. 1B for glycan residue numbering). To generate high mannose-type N-glycans (Fc-Man8), we copied chain A glycan residue 6 for chain A glycan residue 10 and then the glycan A residues 4–10 for chain B glycan residues 4–10. Fc-Man5, Fc-GlcNAc, Fc-N297Q, and Fc/Man8/N297Q were generated by removing glycan residues or mutating N297 to Q297.

To generate simulation input files for each system, we used *Quick MD Simulator* integrated with *Glycan Reader* ^31^ at the CHARMM-GUI website (www.charmm-gui.org)^32^. The TIP3P model was used for explicit water molecules. The cubic system size was determined to have at least 15 Å from the protein in each axis, and 150 mM KCl was added. The system information is given in Table S1. The CHARMM36 force field^33–35^ was used for the proteins and carbohydrates. All calculations were performed at 300 K. The particle mesh Ewald algorithm^36^ was applied to calculate electrostatic forces, and the van der Waals interactions were smoothly switched off at 10–12 Å by a force-switching function^37^. A time step of 2 fs was used in all simulations. Each system was shortly equilibrated in constant particle number, volume, and temperature (NVT) condition with restraints using CHARMM^38^. To assure gradual equilibration of the system, positional restraints for backbone and side chain heavy atoms were applied and the restraint forces were gradually reduced during the equilibration. Additional dihedral angle restraints were applied to restrain all the sugar rings to the pertinent chair conformation. NAMD^39^ was used for additional 10-ns constant particle number, pressure, and temperature (NPT) equilibration without restraints for each system. For NAMD NPT simulation, Langevin coupling coefficient was set to 1 ps^−1^ and a Nosé-Hoover Langevin-piston^40,41^ was used to maintain constant pressure (1 bar) with a piston period of 50 fs and a piston decay of 25 fs.

Each system was further simulated for 2 μs on Anton^42^ using the CHARMM36 force field. The NVT ensemble was used with the temperature maintained at 300 K using the Nosé-Hoover method. The time step was 2 fs and trajectories were saved every 240 ps. The short-range forces and long-range electrostatics were evaluated every 2 fs and 6 fs, respectively. The short-range nonbonded and electrostatic interactions were calculated with a cutoff of 9.52 Å. The long-range electrostatic interactions were calculated using the k-Gaussian Split Ewald method^43^. SHAKE was used to constrain all bonds involving hydrogen atoms. Convergence of the Anton simulations for all systems was checked through the comparison of average RMSD during the first and second halves of the simulations for IgG1 Fc, C′E loop, and glycans (Table S2).

## Electronic supplementary material

Supplementary Information

Video S1. 2-µs simulation movie for Fc-Man8.

Video S2. 2-µs simulation movie for Fc-Man8_R.

Video S3. 2-µs simulation movie for Fc-Man5.

Video S4. 2-µs simulation movie for Fc- GlcNAc.

Video S5. 2-µs simulation movie for Fc- N297Q.

Video S6. 2-µs simulation movie for Fc-Man8/N297Q.
