# Supplementary material for: Effects of N-Glycan Composition on Structure and Dynamics of IgG1 Fc and Their Implications for Antibody Engineering
Source: Sci Rep. 2017 Oct 4;7:12659. doi: 10.1038/s41598-017-12830-5 (PMC5627252; doi:10.1038/s41598-017-12830-5)
Supplement: Supplementary file 1 — Supplementary Information [file 41598_2017_12830_MOESM1_ESM.pdf]

Supplementary Information

Effects of N-Glycan Composition on Structure and Dynamics of IgG1 Fc and Their Implications for Antibody Engineering

Hui Sun Lee and Wonpil Im

Departments of Biological Sciences and Bioengineering, Lehigh University, 111 Research Drive, Bethlehem, PA 18015, USA

Table S1. MD simulation system information.

System name	# water	# ions (K^+, Cl^-)	# total atoms
Fc-Man8	46,320	132, 132	146,672
Fc-Man5	46,370	132, 132	146,696
Fc-GlcNAc	46,494	133, 133	146,806
Fc-N297Q	46,531	133, 133	146,869
Fc-Man8/N297Q	45,247	129, 129	143,228

Table S2. Convergence check of 2-μs Anton simulations through the comparison of average RMSD (Å) during the first and second halves of the simulations. The RMSD was calculated using CA atoms for IgG1 Fc and all heavy atoms for C′E loop and glycans with respect to the initial structure.

System name	IgG1 Fc	C′E loop		Glycan	
		$C\gamma 2_A$	$C\gamma 2_B$	$C\gamma 2_A$	$C\gamma 2_B$
Fc-Man8	4.5±0.9/3.9±0.6	5.2±1.7/6.4±0.5	3.7±1.2/4.4±0.8	13.0±5.4/15.7±7.1	18.6±9.9/18.5±4.6
Fc-Man8_R	5.1±1.0/5.9±0.9	2.8±0.8/2.8±0.8	3.1±0.9/2.9±1.0	6.5±1.9/8.3±2.5	8.7±2.4/8.8±1.8
Fc-Man5	4.9±0.7/5.1±0.4	3.7±1.0/6.0±1.2	5.9±1.5/5.4±1.4	8.0±3.3/16.8±4.5	10.1±1.7/16.1±5.5
Fc-GlcNAc	4.3±0.9/4.4±0.9	3.5±0.8/3.3±0.7	4.0±0.5/4.1±0.4	4.0±0.7/4.5±0.6	5.9±0.5/4.5±0.6
Fc-N297Q	4.9±0.9/5.1±0.9	4.0±1.1/4.3±0.8	7.7±1.5/7.4±1.0		
Fc-Man8/N2979	4.8±1.0/4.8±0.6	3.2±0.8/3.1±0.7	4.6±0.8/4.7±1.1	11.2±5.1/10.4±3.9	

Figure S1. Structure of IgG1 Fc in complex with FcγRIIIa. (A) IgG1 Fc (green) and FcγRIIIa (orange) are shown in cartoon representation (PDB: 3sgk). Glycans are represented in stick. Potential hydrogen bonds are shown as dashed red lines. (B) Structure comparison of GlcNAc1 between the MD initial structure and IgG1 Fc/FcγRIIIa crystal structure.

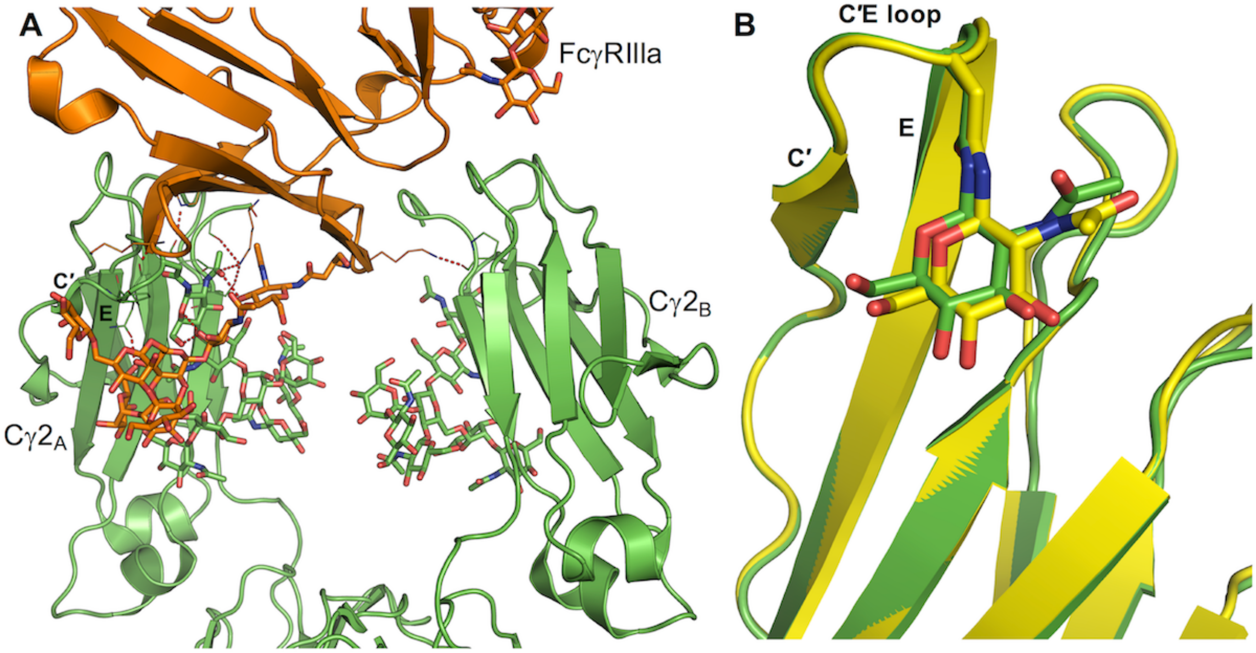

Figure S2. β-strand secondary structure time-series of C′ strand for polypeptide A (blue) and B (red).

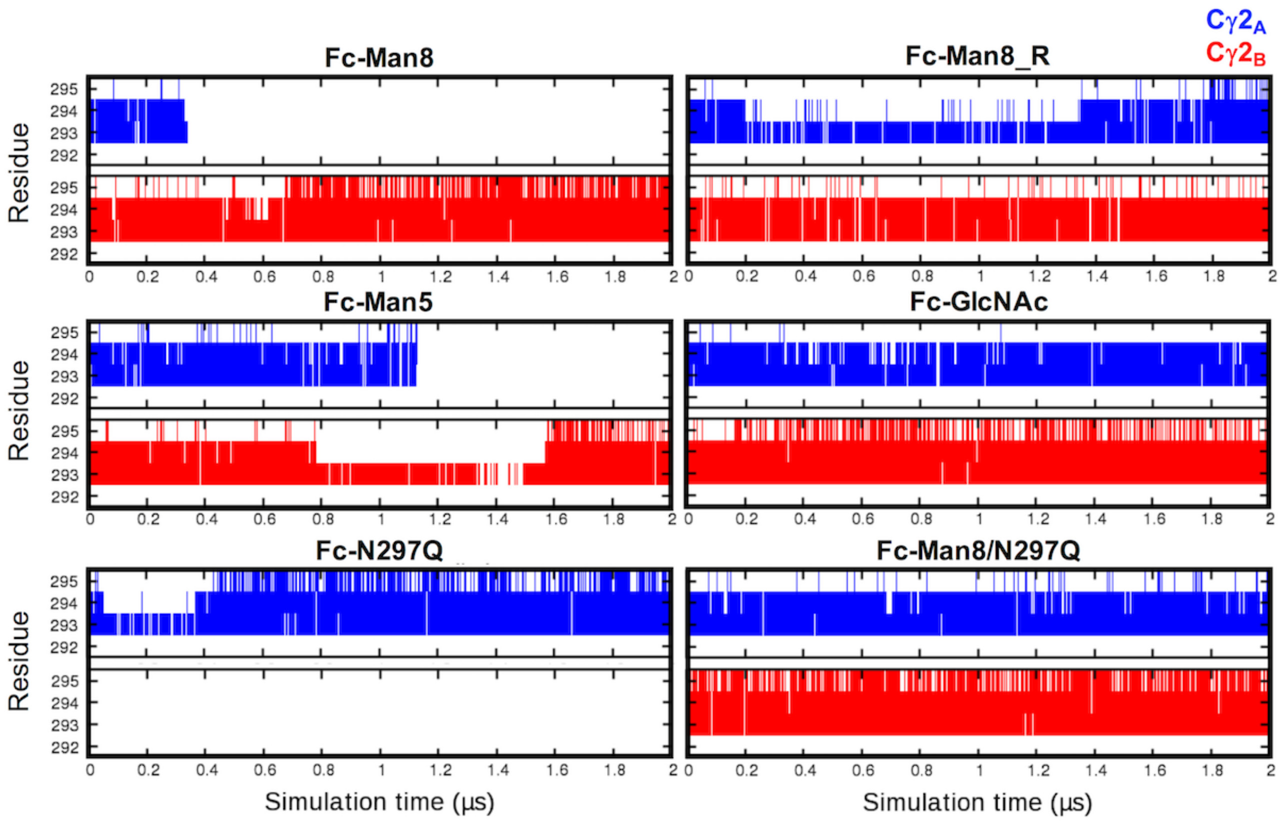

Figure S3. Schematic illustration of definition of points for Cγ2-Cγ3 angles (*θ*) and dihedral angles (*ϕ*) calculation. Three points for the angle calculation were defined from the Cα atoms of residues Y300, M428, and Q362. Four points for the dihedral angle calculation were defined from the Cα atoms of residues Y300, Y319, M428, and Q362.

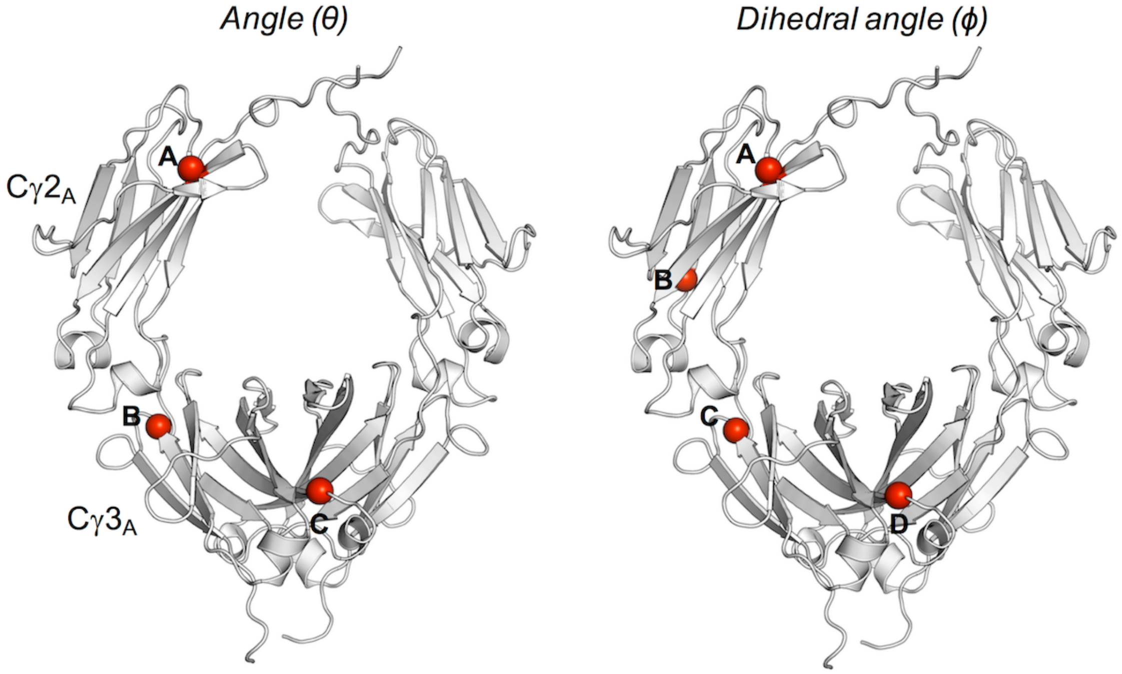

Figure S4. Distributions of Cγ3-Cγ3 angles (θ) and dihedral angles (ϕ). Three points for the angle calculation were defined from the Cα atoms of residues M428 (chain A), and Q362 (chain A), and M428 (chain B). Four points for the dihedral angle calculation were defined from the Cα atoms of residues M428 (chain A), Q362 (chain A), Q362 (chain B), and M428 (chain B). The magenta lines in each plot correspond to the angle and dihedral angle in the initial structure.

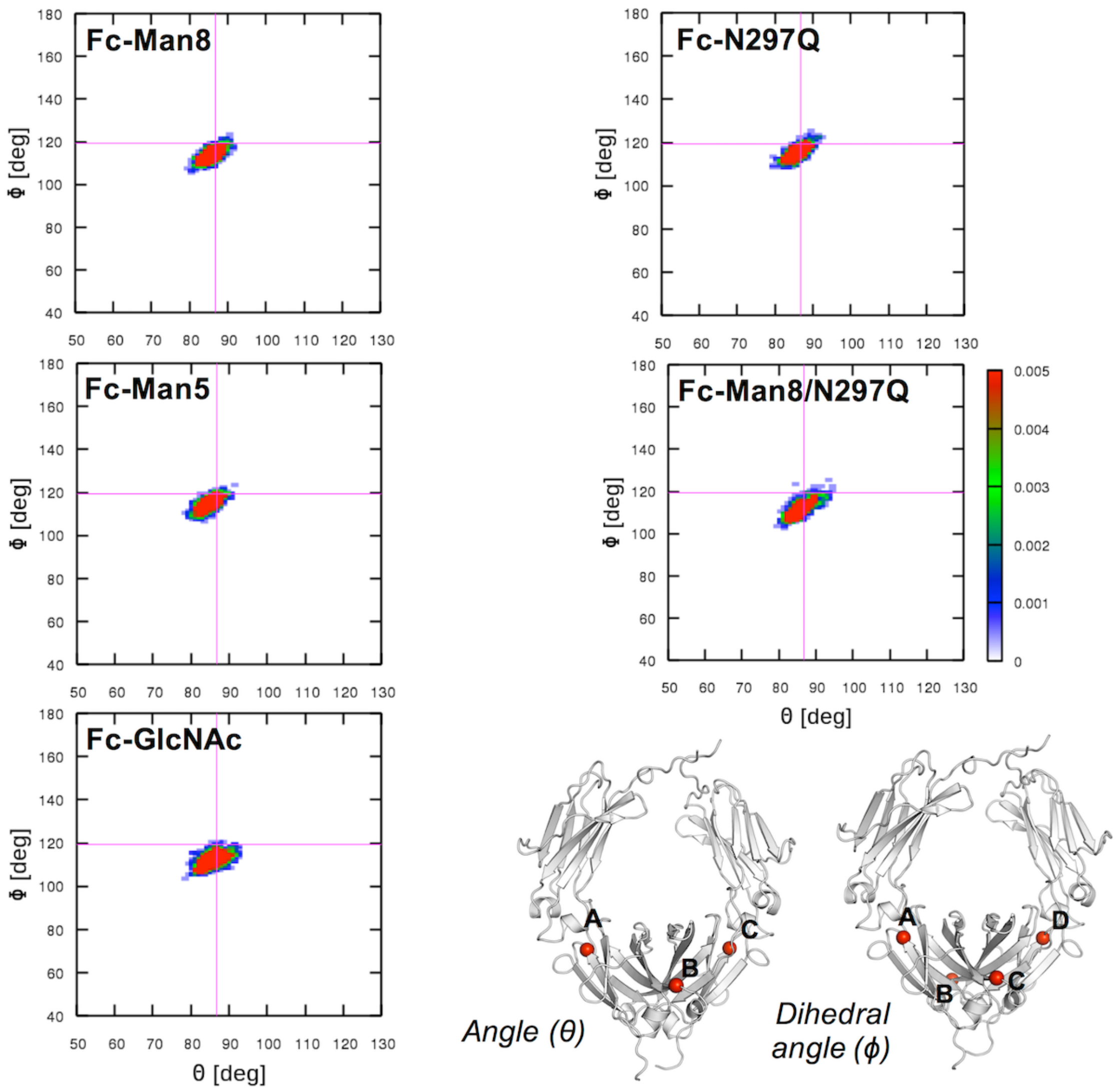

Figure S5. N-glycan motions and Cγ2-Cγ3 orientations in Fc-Man8. (A) Time-series of minimum distances between any heavy atoms of glycan termini (residues 6, 7, and 10) and any protein heavy atoms. (B) Time-series of Cγ2-Cγ3 angles (θ) (blue) and dihedral angles (ϕ) (red). The correlation coefficient values between the distance time-series and angle time-series are also shown on the plots.

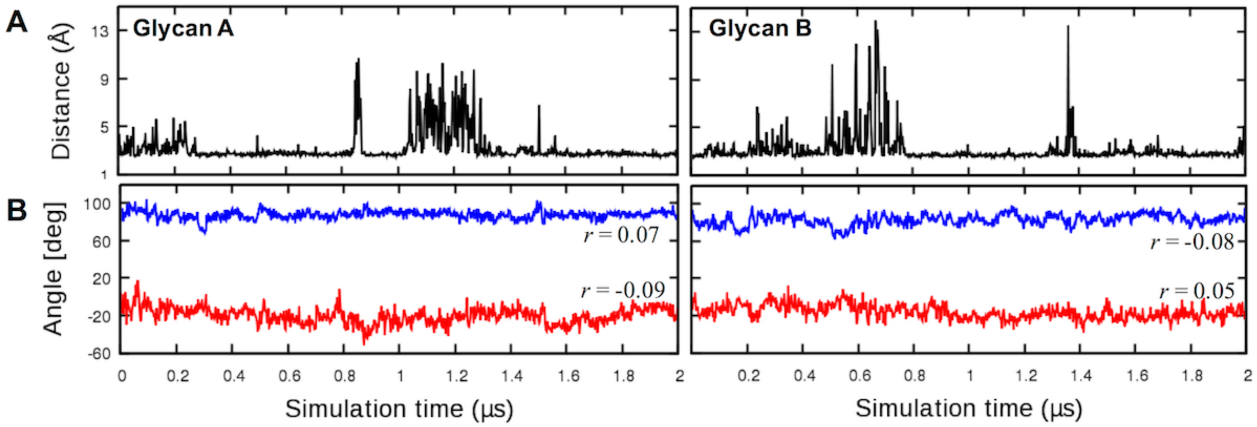

Video S1. 2-μs simulation movie for Fc-Man8.

Video S2. 2-μs simulation movie for Fc-Man8_R.

Video S3. 2-μs simulation movie for Fc-Man5.

Video S4. 2-μs simulation movie for Fc- GlcNAc.

Video S5. 2-μs simulation movie for Fc- N297Q.

Video S6. 2-μs simulation movie for Fc-Man8/N297Q.
